# Atomization Characteristics of Hydrogen Peroxide Solutions in Electrostatic Field

**DOI:** 10.3390/mi13050771

**Published:** 2022-05-13

**Authors:** Xuefeng Huang, Ling Sheng, Yibin Lu, Shengji Li

**Affiliations:** 1 Institute of Energy, Department of Physics, Hangzhou Dianzi University, Hangzhou 310018, China; xuefenghuang@hdu.edu.cn (X.H.); 201070068@hdu.edu.cn (L.S.); bylielu@163.com (Y.L.); 2 College of Materials and Environmental Engineering, Hangzhou Dianzi University, Hangzhou 310018, China

**Keywords:** electrostatic atomization, hydrogen peroxide, surface tension, viscosity, concentration effect

## Abstract

Hydrogen peroxide (H_2_O_2_) can be considered as a sterilant or a green propellant. For a common use in industrial application, spray is an effective method to form fine H_2_O_2_ droplets. In this paper, electrostatic atomization based on the configuration of needle ring electrodes is proposed to produce H_2_O_2_ spray by minimizing its effective surface tension. The breakup performances of H_2_O_2_ ligaments can be improved by increasing the electric field intensity, reducing the nozzle size, and adjusting suitable volume flow rate. The smallest average diameter of breakup droplets for 35 wt. % concentration H_2_O_2_ solution reached 92.8 μm under optimum operation conditions. The H_2_O_2_ concentration significantly influenced the breakup performance owing to the concentration effect on comprehensive physical properties such as density, surface tension, viscosity, and permittivity. The average diameters of breakup droplets decreased with decreasing H_2_O_2_ concentration. At 8 wt. % concentration, the average breakup droplet diameter was reduced to 67.4 μm. Finally, electrostatic atomization mechanism of H_2_O_2_ solution was analyzed by calculating dimensionless parameters of *Re*, *We*, and *Oh* numbers with the combination of the operation conditions and physical properties for in-depth understanding the breakup behaviors. The calculation showed that the minimum average diameter of breakup droplets was obtained at 8 wt. % concentration at the investigated range of H_2_O_2_ concentration, which kept in agreement with the experimental results.

## 1. Introduction

Owing to the active chemical properties and strong oxidation, H_2_O_2_ has been widely used as the sterilant in sterilizers for medical instruments [1,2,3], also for microbial reduction in NASA planetary protection [4]. H_2_O_2_ is a slightly light blue gas, which can be miscible with water in any proportion. Different concentrations of H_2_O_2_ solutions are utilized in different fields for specific purposes. Generally, low-concentration H_2_O_2_ solution of 3 wt. % is mainly used for bacteriostatic disinfection of skin, oral mucosa, and wound surfaces. H_2_O_2_ solutions of 25~50% concentration are applied in the field of industrial disinfection, such as GMP standard workshops, medical precision instruments and equipment, pipe tuyere, pulp, fabric bleaching, etc. [5]. Its nontoxic, harmless, and pure characteristics make it an ideal medical disinfectant. Food-grade H_2_O_2_ solution is used in aseptic dairy product packaging and aquatic product disinfection.

Highly concentrated H_2_O_2_ solution is considered as a nontoxic, noncarcinogenic, and green propellant [6,7], as well as a liquid oxidizer [8,9] for propulsion systems, owing to being able to decompose exothermically into steam and oxygen. Nagiev et al. [10] and Quinn et al. [11] conducted the ignition test of 90% high-concentration H_2_O_2_ and hydrocarbon mixed fuel and concluded that the ignition delay reached 16 ms. Since then, a propellant named D- α code for the external 3.8 kN thrust has been developed, and its ignition delay time was shortened to 7.9 ms. H_2_O_2_ was preferentially adopted by NASA as the fuel in the rocket-based combined cycle-propulsion system (RBCC) because of its high combustion propulsion efficiency and high ignition characteristic speed [12,13]. A three-strand injection device was adopted inside the engine to strictly control the composition and fuel ratio. The main components of the propellant contain H_2_O_2_ with a concentration of more than 89%. After ignition, the peak pressure in the combustion propulsion chamber reached 10 MPa, and the working time limit of flame maintenance was up to 6 min, which provided strong endurance for propulsion, and has successfully completed dozens of engine tests [14]. In the recent FP7 research program in Europe, H_2_O_2_ solution was used as an advanced green space propellant (grasp) in the development of rocket thrusters [15,16,17]. H_2_O_2_ has strong oxidation characteristics similar with other liquid oxidants, such as nitrogen oxide, nitric acid, and liquid oxygen. It can be utilized as double-based propellant or mixed propellant with other oxidants. The combination of H_2_O_2_ solution of 87.5% with kerosene or ethanol has proved to be the best choice of green propellant.

When used as a sterilizing agent, H_2_O_2_ mostly needs to be atomized into small droplets and/or vaporized. After evaporation, water vapor and H_2_O_2_ gas fill with the chamber, and water vapor tends to condensate on drops in the chamber, which interferes with the sterilizing action of gas-phase H_2_O_2_. The water condensation will also prejudice the observation. When used as a propellant, the H_2_O_2_ solution forms a droplet group by atomization before combustion. The uniform dispersion of atomization determines the sufficient mixing degree of different fuels, combustion efficiency, and jet flame stability in the process of combustion. These drive us to find an alternative atomization method for the H_2_O_2_ solution.

Electrostatic atomization, known as electrospray or electrohydrodynamic atomization, is a method in which the fluid breaks up into fine charged droplets with diameters between ten and several hundred micrometers and a relatively narrow size distribution. By contrast with other atomization techniques, electrostatic atomization has some advantages, namely relative ease of droplet generation, great control of droplet transport, ability to avoid coalescence of droplets due to electric charge of the same polarity on the droplets, enhanced adhesion, deposition, and so on [18,19].

The charges significantly affect the particle-size distribution and fragmentation size of droplets, providing better atomization characteristics, mixing ratio, uniformity, permeability, and surface adhesion. Therefore, to charge the solution as soon as possible in a short time, the charge is injected into the liquid through an external electrostatic device. The common charging methods are corona charging, induction charging, and contact charging [20]. Hara et al. [21] studied the current waveform of corona charging and the relationship between specific charge and droplet size. The maximum charge ratio of distilled water could be confirmed to be 5 × 10^−3^ C/kg, and the duration of the current was closely related to the force that determines the deformation of water droplets. Maski et al. [22] explored the induced charging performance of liquid in 4 kV voltage and low flow rate (30, 45, 60 mL/min) mode. The combination of low flow rate and inductive charging characteristics could increase the charging power of the electrostatic spray and the spray electrification capacity. Chen et al. [23] measured the droplet size, velocity, and gas temperature in a flame by means of a special electrostatic needle spray device. According to the flame structure caused by the countercurrent diffusion of monodisperse heptane, the droplet had evaporated completely before the direct interaction with the flame, and the droplet diffusion surface was large. The atomization effect was infinitely close to the pure gasification diffusion. Kreitzer et al. [24] demonstrated that conical electrodes were used to generate spray to reduce heat transfer and improve the cooling system. When the charging voltage exceeded 15 kV, a great change in the spray flow pattern was observed. 

Among the patterns of charging, contact charging possesses higher efficiency. In addition, a difficulty of low breakup efficiency needs to be solved due to relatively high surface tension of H_2_O_2_ solution. Therefore, to improve the breakup performance of H_2_O_2_ solution, in this study, we propose to investigate the electrostatic atomization characteristics of H_2_O_2_ solution by contact charging pattern based on the needle ring electrodes. The objectives of present work will be involving: (1) to investigate the effects of the applied electric field voltage, volume flow rate, nozzle size, and concentration on the electrostatic breakup and droplet formation of H_2_O_2_ jet mode in detail, and then to achieve the optimized electrostatic atomization parameters; (2) to reveal the electrostatic atomization mechanism of H_2_O_2_ by a more in-depth analysis of dimensionless parameters, including Reynolds number, Weber number, and Ohnesorge number.

## 2. Materials and Methods

### 2.1. Materials

Two concentrations of H_2_O_2_ solutions (8 wt. % and 35 wt. %) were provided from Zhejiang TAILIN Bioengineering Co., Ltd., Hangzhou, China. The H_2_O_2_ solution of 35 wt. % was diluted by adding the deionized water to prepare different concentrations of H_2_O_2_ solutions to perform the concentration effect test, i.e., 15 wt. %, 20 wt. %, 25 wt. %, and 30 wt. %, respectively.

**Figure 1:**
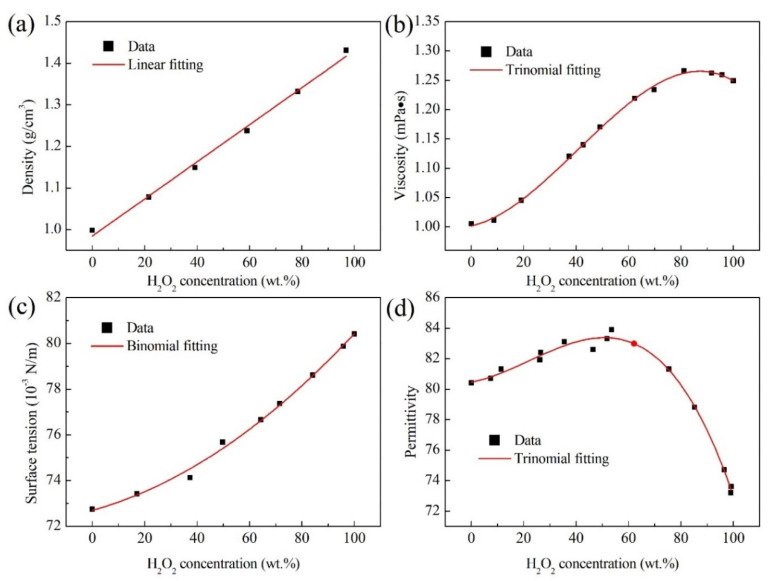
The physical properties of H_2_O_2_ solutions at the room temperature of ~20 °C. (**a**) Density; (**b**) Viscosity; (**c**) Surface tension; (**d**) Relative permittivity.

At the room temperature of ~20 °C, the physical properties of H_2_O_2_ solutions including the density, surface tension, viscosity, and permittivity are shown in Figure 1 [25]. They were measured by a dilatometer, capillary rise method, Ostwald viscometer, and a resonance method, respectively. Their relations with H_2_O_2_ concentration (*φ*) are listed into Table 1. The density has a linear function with the concentration at the slope of 0.0044. The surface tension is a binomial function as the concentration. The viscosity and permittivity vary with a trinomial function with the concentration. In the range of 0~35 wt. %, the four physical properties of H_2_O_2_ solutions increase with increasing H_2_O_2_ concentration. 

The physical properties of H_2_O_2_ solutions also depend on temperature. Therefore, during the experiments, it is necessary to control the stability of the laboratory-room temperature to prevent the volatilization of the solution.

### 2.2. Experimental Setup

**Figure 2:**
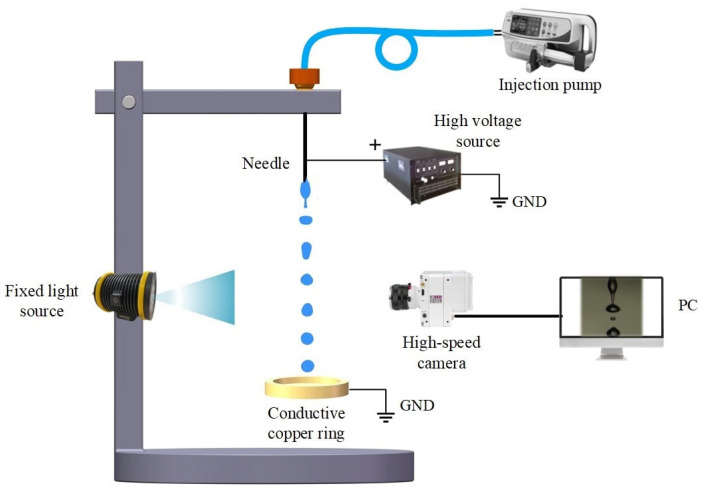
Schematic diagram of experimental setup.

Figure 2 shows the schematic diagram of experimental setup for electrostatic atomization, including liquids and feeding system, electrospray high-voltage system, and illumination and visual imaging system. All electrostatic atomization experiments were conducted under atmospheric pressure and room temperature.

#### 2.2.1. Liquids and Feeding System

The H_2_O_2_ solutions were firstly stored in a 50 mL syringe, and then pumped into atomization nozzles to produce a ligament (jet) through a Teflon-coated pipe. Twenty atomization nozzles composed of silica glass (Table 2) were alternatively used in this work. The inner diameters of these nozzles range from 0.16 mm to 3.0 mm. The thickness of the nozzles along with radial direction and the length along with axial direction are 0.075~0.30 mm and 25 mm, respectively. The nozzles were vertically held on a fixed stage. To prevent the formation of bubbles, H_2_O_2_ solutions were sonicated for a few minutes before loading into the syringes. A syringe micropump (TYD02, Lead Fluid Technology Co., Ltd., Baoding, China) was used to control the volume flow of H_2_O_2_ solutions with an accuracy of ±2%.

#### 2.2.2. Electrospray High-Voltage System

The needle ring electrodes composed of copper material were used to charge the H_2_O_2_ solutions based on the contact-charging pattern. The needle electrode was connected to the positive pole of a high-voltage power supply (73030P, General High Voltage Ind. Ltd., Bridgnorth, UK), the ring electrode was grounded, and the ring center was concentric with the needle. The distance between the end of the needle and the ring electrodes was fixed at 3.5 cm to keep the same electrostatic field condition. The high-voltage source was exerted on the needle ring electrodes to produce a high electric-field intensity. The liquid flowed out through the nozzles, formed a group of droplets after electrostatic atomization, and fell through the lower ring electrode.

#### 2.2.3. Illumination and Visual Imaging System

The electrostatic atomization process was visualized by using a high-speed camera (M310, Phantom Inc., San Francisco, CA, USA) with a high-resolution lens (Nikon, Tokyo, Japan). A LED light source with uniform backlight, placed on the opposite position of the high-speed camera, was used to provide view field illumination with obvious contrast under the condition of large aperture and short-time exposure. The recording frame rate of high-speed camera was set to 15,000 fps with the image resolution of 512 × 384.

The characteristic parameters of atomized droplets of H_2_O_2_ solutions, such as the number of droplets and particle-size distribution, were analyzed by digital imaging treatment described in our previous work [26,27]. 

## 3. Results and Discussion

In an electrostatic field, when a liquid ligament is exerted by the electrical stress that exceeds surface tension, the ligament breaks up into droplets due to instability [28]. The electric field intensity, nozzle size, volume flow rate, and H_2_O_2_ concentration influence electrostatic breakup characteristics of H_2_O_2_ ligaments. Therefore, the effects on the atomization characteristics were experimentally evaluated to deeply recognize electrostatic breakup and droplet formation.

### 3.1. Effect of Electric-Field Voltage on the Breakup Performance

**Figure 3:**
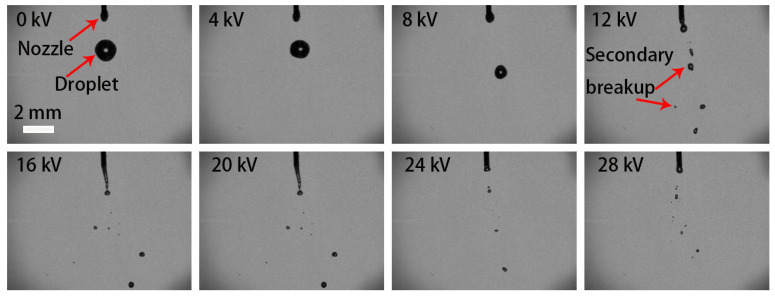
Pictures of electrostatic breakup of H_2_O_2_ ligaments and droplet formation at different electric field voltages (21G nozzle, flow rate of 400 mL/h, 35 wt. % concentration).

The nozzle 21G was installed, and the flow rate was set to 400 mL/h. The variation of H_2_O_2_ ligaments with electric-field voltage is shown in Figure 3. As the voltage was below 10 kV, only an individual mother droplet dropped from the nozzle outlet; the charge accumulated on the droplet surface was less, and the electrostatic force could not balance the surface tension to break up the mother droplet. When the voltage was adjusted to 10–28 kV, the H_2_O_2_ ligaments underwent catastrophic fragmentation. It suggests that the electrostatic force of charged droplets exceeds the surface tension, resulting in secondary breakup.

**Figure 4:**
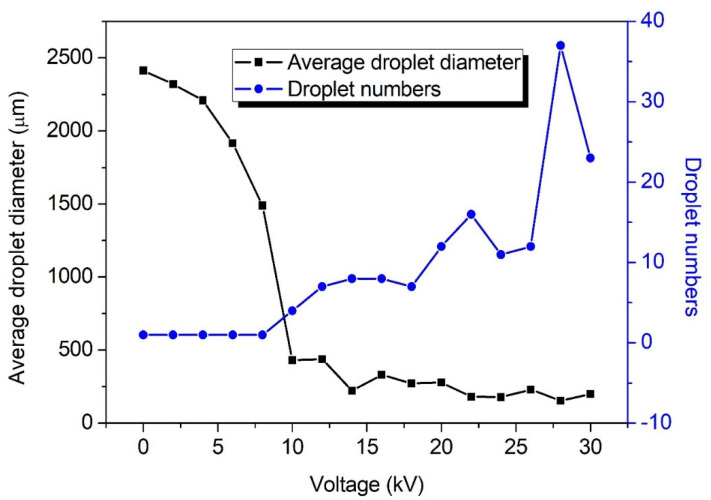
The numbers of breakup droplets and the average droplet diameters at different voltages (21G nozzle, flow rate of 400 mL/h, 35 wt. % concentration).

The number of breakup droplets and the average droplet diameters were statistically analyzed and illustrated in Figure 4. It was found that the number of droplets rose sharply for the first time at 10 kV. This is because smaller satellite droplets were produced during the pinch-off of the main droplets from the ligaments. With increasing the voltage, about 14 small droplets could be split out after a periodic fall off. At 28 kV, the number of droplets rose rapidly for the second time to 37. At 30 kV, the number of droplets was quite unstable due to significant air ionization. Although the number of droplets decreased, it still exceeded the average level, with a gap of about 10 droplets.

The average sizes of breakup droplets varied with the voltages (Figure 4). At relatively low voltage (below 10 kV), the sizes of most of droplets were greater than 1000 μm, twice or more than the nozzle inner diameter. The ligament fell off in the form of a main droplet and did not form effective fragmentation. Under the condition of medium voltage, i.e., 10–20 kV, with the emergence of small satellite droplets, the average size began to decline, especially at 10 kV. At high voltage of over 22 kV, the sizes of breakup droplets below 100 μm gradually began to appear. The proportion of smaller droplets below 100 μm enhanced with increasing the voltage. At 28 kV, the proportion was the highest and the average droplet size reached the minimum value of 154.8 μm. The sizes of broken droplets mainly distributed in 51.2–194.5 μm.

**Figure 5:**
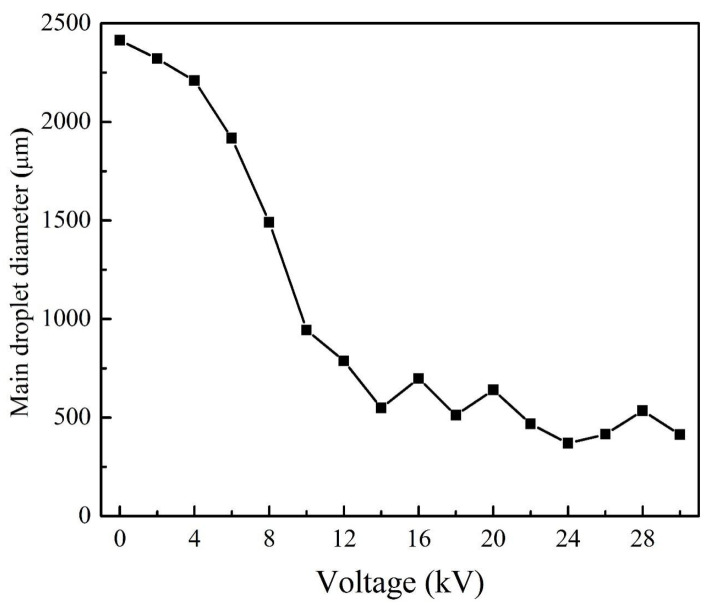
The average diameters of main droplets at different voltages (21G nozzle, flow rate of 400 mL/h, 35 wt. % concentration).

It can be found that the sizes of the main droplets (mother droplets) can be changed by changing the voltage, and their size distribution is the key factor to evaluate the atomization performance. The main droplet size trend, shown in Figure 5, demonstrated that the sizes varied from the initial 2413.5 μm and gradually decreased to 369.7 μm, and then remained flat. It was also observed that the main droplets generated periodically in the droplet group and gradually decreased and became stable as the voltage was enhanced.

At 28 kV, the atomization performance kept the best (Figure 4). Therefore, the voltage of 28 kV was selected for performing subsequent experiments to maintain good breakup performance.

### 3.2. Effect of Volume Flow Rate on the Breakup Performance

**Figure 6:**
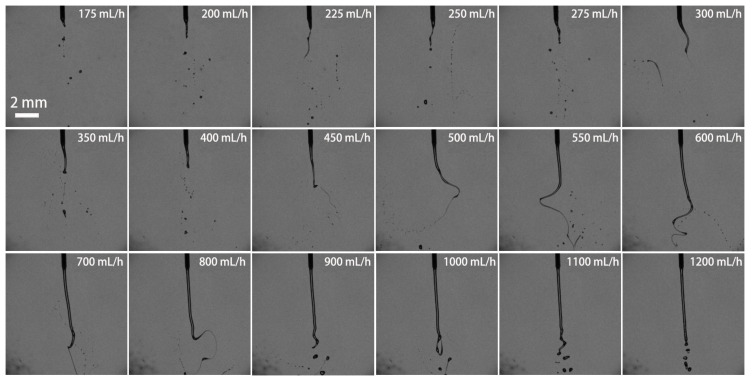
Pictures of electrostatic breakup of H_2_O_2_ ligaments and droplet formation at different volume flow rates (21G nozzle, voltage of 28 kV, 35 wt. % concentration).

Figure 6 demonstrates the variation of H_2_O_2_ ligaments with the volume flow rate. When the flow rate was set to below 100 mL/h, main droplets and occasional satellite droplets formed at the nozzle outlet. The number of droplets was extremely tiny. With increasing the flow rate, at 100–550 mL/h, it can be found that the jet formed at the nozzle outlet and began to swing under the action of electrostatic force, forming a slender liquid braid with a cone angle at the end. The swing amplitude and length of the braid became larger as the flow rate was enhanced further. As a result, the electric-field force made the long braid break up and split, producing quantities of droplets. When the flow rate was increased to 600 mL/h, the jets became longer. The electrostatic force produced at 28 kV difficultly made the jets break up. The swing amplitude of the long braid gradually attenuated and remained stable. Finally, under an ultrahigh flow rate of over 1000 mL/h, the filaments only break up into some large droplets. It is worthy to note that the formation of the liquid braid possibly resulted from the influence of extensional viscosity. When the liquid braid suffered from both the electrostatic stress and the viscous stress, the phenomena of the spinning of the ligament and the droplets on a string were observed. 

**Figure 7:**
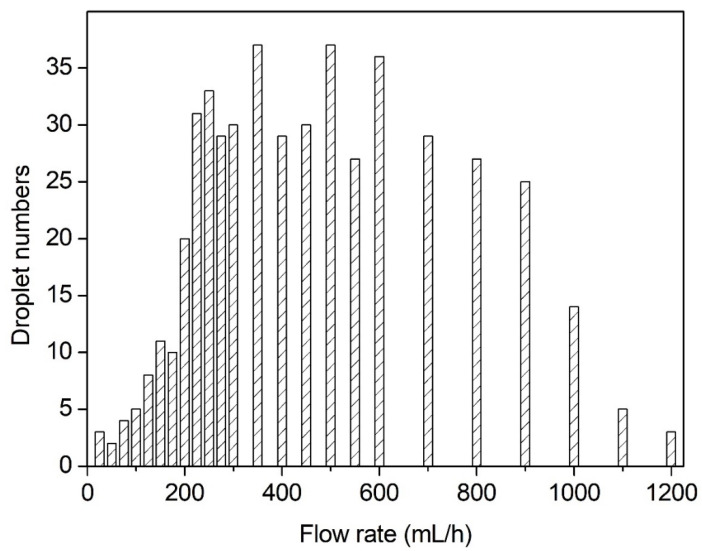
The numbers of breakup droplets at different volume flow rates (21G nozzle, voltage of 28 kV, 35 wt. % concentration).

The number of breakup droplets varied with the volume flow rate ranging from 25–1200 mL/h, as shown in Figure 7. If the flow rate was in the range of 25–500 mL/h, the number of droplets increased due to the increase in the flow rate. The first obvious sharp rise was located at 175 mL/h. When at 500 mL/h, the number of droplets reached a peak of 37. When the flow rate ranged from 500 mL/h to 1200 mL/h, the number of droplets began to decline continuously. The first obvious drop was at 1000 mL/h. At the ultrahigh flow rate of 1200 mL/h, the breakup became more difficult. 

**Figure 8:**
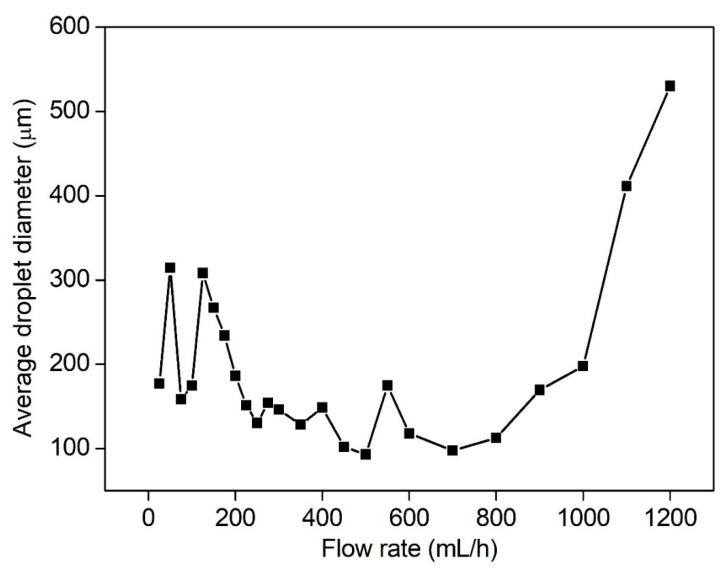
The average diameters of breakup droplets at different volume flow rates (21G nozzle, voltage of 28 kV, 35 wt. % concentration).

At the flow rate of 25–175 mL/h, the average diameters of breakup droplets ranged 158–308 μm (Figure 8). The fluctuation possibly resulted from the unstable flow of the micropump at low flow rates. At the flow rate of 175–700 mL/h, the average diameters began to decrease and tended to be flat. The smallest average diameter was 92.8 μm at 500 mL/h. At the flow rate of 700–1200 mL/h, the average diameters of breakup droplets gradually increased. It does not mean that the higher the number of breakup droplets, the lower the average diameter of droplets. In the case of relatively low flow rate (0–200 mL/h), the size distribution of most breakup droplets was 200–400 μm. The proportion of droplets below 100 μm was relatively low, and the size-distribution spectrum gradually widened with the increase in the flow rate. At medium and high flow rates of 200–500 mL/h, the size of breakup droplets was mainly distributed in 50–200 μm, and the size-distribution spectrum was narrowed. For the high flow rate of 500–1000 mL/h, the proportion of droplets in 50–100 μm showed a downward trend, resulting in the size distribution beginning to widen. At the ultra-high flow rate of 1000–1200 mL/h, large droplets could not be broken up effectively and the size distribution became wider.

To sum up, when the nozzle size and the applied voltage were fixed, there was a critical value of volume flow to make the droplet diameter smallest. In the case of the 21G nozzle and 28 kV, the critical volume flow rate was 500 mL/h.

### 3.3. Effect of Nozzle Size on the Breakup Performance

**Figure 9:**
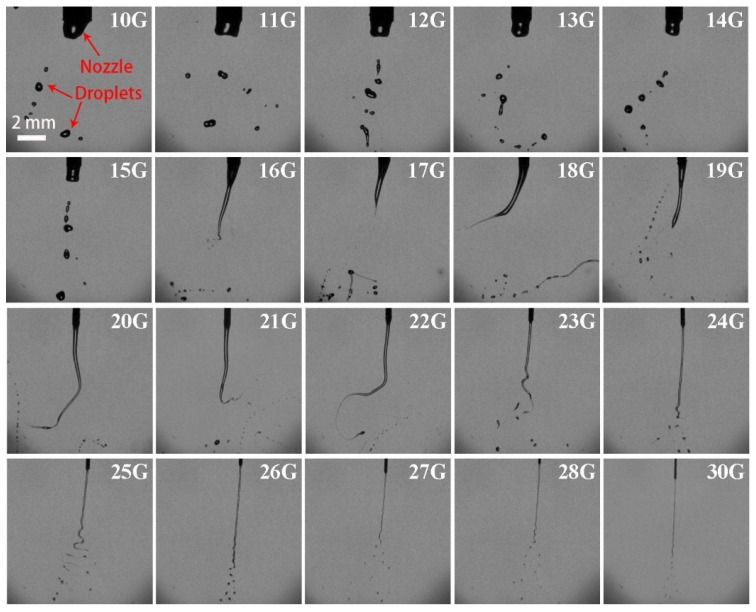
Pictures of electrostatic breakup of H_2_O_2_ ligaments and droplet formation at different nozzle sizes (flow rate of 500 mL/h, voltage of 28 kV, 35 wt. % concentration).

**Figure 10:**
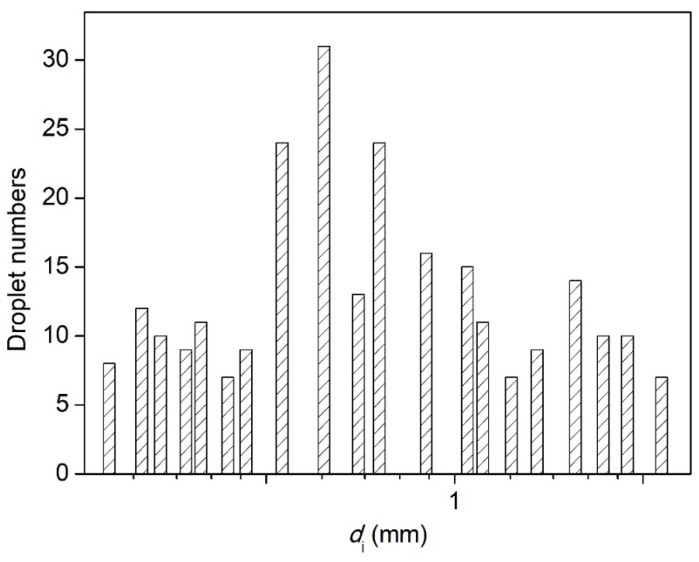
The droplet numbers of H_2_O_2_ ligaments electrostatic breakup at different nozzle sizes (flow rate of 500 mL/h, voltage of 28 kV, 35 wt. % concentration).

**Figure 11:**
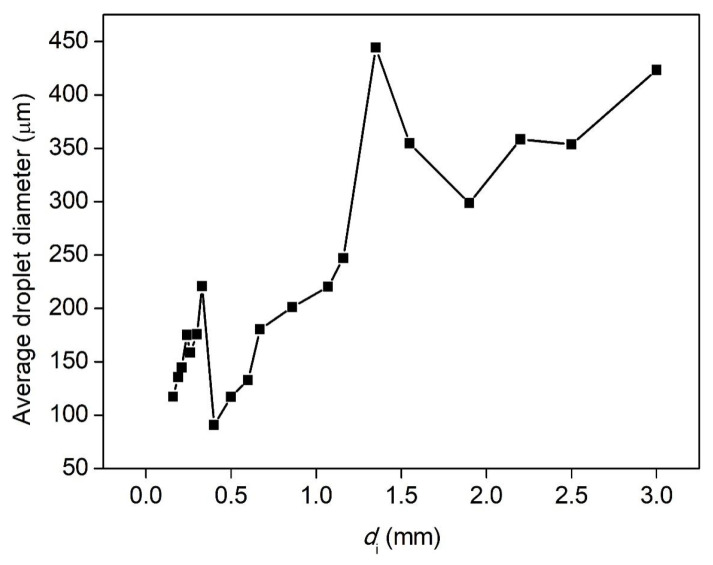
The average diameters of H_2_O_2_ ligaments electrostatic breakup at different nozzle sizes (flow rate of 500 mL/h, voltage of 28 kV, 35 wt. % concentration).

The breakup of ligaments through the nozzles with different sizes, at the volume flow rate of 500 mL/h and the applied voltage of 28 kV, is shown in Figure 9. The droplet numbers and the average diameters are illustrated in Figure 10 and Figure 11. The histogram of the droplet numbers shows that the first lifting occurred at 22G and 23G nozzles (inner diameter of 0.33 mm and 0.40 mm, respectively). The number of droplets reached the maximum for the 21G nozzle. With the decrease in the nozzle size, the droplet number showed a downward trend. The smaller the nozzle sizes were, the longer the length of ligaments became. It resulted in weak and ineffective charging, and the fragmentation became difficult. Therefore, at the volume flow rate of 500 mL/h, many more droplets could be produced by selecting the 19G–22G nozzles (inner diameter of 0.40–0.67 mm). 

The average diameters of breakup droplets totally showed a nonmonotonic downward trend with decreasing the nozzle size, but there were two significant leaps at 13G–15G and at 22G–23G, respectively. For 23G–30G (inner diameter 160–330 μm), the atomization cone angle increased. The droplet-size distribution of 23G was wider than 22G. To obtain optimum breakup performance, in this case, 20G–22G nozzles (inner diameter of 0.4–0.6 mm) need to be installed. The average diameters of droplets reached the minimum value of 90.7 μm with good uniformity and dispersion at 22G.

### 3.4. Effect of H_2_O_2_ Concentration on the Breakup Performance

**Figure 12:**
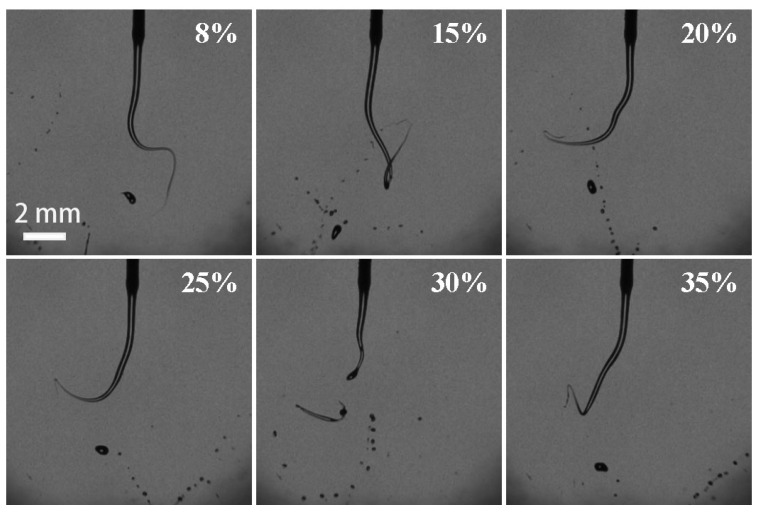
Pictures of electrostatic breakup of H_2_O_2_ ligaments and droplet formation at different H_2_O_2_ concentrations (21G nozzle, flow rate of 500 mL/h, voltage of 28 kV).

**Figure 13:**
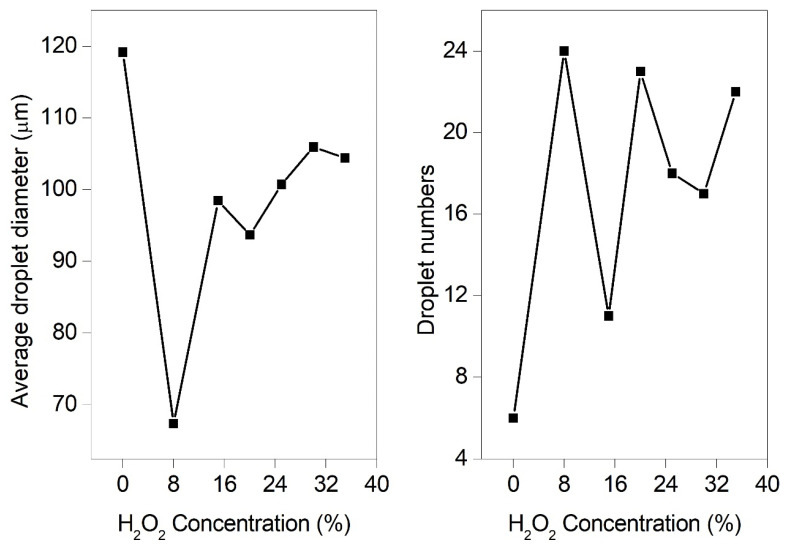
The average droplet diameters and the numbers of breakup droplets at different H_2_O_2_ concentrations (21G nozzle, flow rate of 500 mL/h, voltage of 28 kV).

By comparing the breakup performance of H_2_O_2_ solutions at different operation conditions, it was found that one of the optimum parameters were 500 mL/h for the volume flow rate, 28 kV for the electrostatic voltage, and 0.5mm (21G) for the nozzle size, respectively. Therefore, the effect of H_2_O_2_ concentration on the breakup performance was experimentally performed at the optimum operation conditions. Figure 12 demonstrated the representative breakup snapshots of H_2_O_2_ solutions with different concentrations. Their average diameters and the numbers of breakup droplets were statistically illustrated in Figure 13. The H_2_O_2_ solution of zero concentration (0 wt. %) means that it is pure deionized water. The results show that the breakup fine droplets of deionized water were mainly in the size range of 104.1–135.7 μm. The droplet-size distribution was uniform, and the average particle size was 119.2 μm. Compared with deionized water, for H_2_O_2_ solution of 8 wt. % concentration, the average diameter was 67.4 μm. The increase in H_2_O_2_ concentration led to the increase in the average droplet diameter. At the concentration in the range of 8–20 wt. %, the growth rate of the average droplet diameters was significantly faster than that in the range of 20–35 wt. %. At high concentration of 30–35 wt. %, the maximum average diameters of breakup droplets were over 100 μm; however, this was lower than that of pure deionized water. The slight oscillation of diameters of breakup droplets and droplet numbers possibly resulted from the instability of the volume flow rate.

### 3.5. Mechanism Analysis on Electrostatic Atomization of H_2_O_2_ Solution

As the liquid is extruded from a nozzle by pressure, a Taylor cone is formed and a ligament (jet) with unstable surface wave perturbation is produced [28]. If the external force exceeds the surface tension, the ligament will break up into fine droplets. In this work, to produce ultrafine uniformly distributed droplets, the needle ring electrodes were applied to ligaments, and the electrostatic force resulted from the strong electric-field intensity exerted on the ligaments and made them catastrophically break up into fine droplets.

As mentioned above, the size of breakup droplets relates to the physical properties (density, viscosity, surface tension, permittivity, and concentration etc.) of H_2_O_2_ solutions and operation conditions (applied voltage, nozzle size, and volume flow rate). To analyze the breakup behavior of H_2_O_2_ solutions, the dimensionless parameters that define the ligaments breakup are the Reynolds number (*Re*), the Weber number (*We*), and the Ohnesorge number (*Oh*) without electric field force [29]:

(1) $$Re=\frac{\rho ud_{i}}{\mu },We=\frac{\rho u^{2}d_{i}}{\gamma },Oh=\frac{\mu }{\sqrt{\rho \gamma d_{i}}}$$

where *u* is the velocity of flow, relating with the volume flow rate (*Q*) and the nozzle size (*d*_i_). They follow the function of $u=4Q/\left(\pi d_{i}^{2}\right)$.

For the cases in Section 3.2, as the volume flow rate was enhanced from 25 mL/h to 2000 mL/h, the *Re* number increased linearly, from 14.2 to 1135.5. This suggests that the breakup of the H_2_O_2_ ligaments generally exhibited a laminar regime. The *We* number increased binomially with the volume flow rate from 0.008 to 47.9. The *Oh* number kept constant, which was independent of the volume flow rate. For the cases in Section 3.3, as the nozzle size was reduced from 3.0 mm to 0.16 mm, the *Re* number was inversely proportional to the nozzle size, ranging from 47.3 to 887.1. The *We* number was a function of the negative cubic of the nozzle size and increased from 0.014 to 91.3. The *Oh* number increased from 0.002 to 0.01. For the cases in Section 3.4, the *Re* and *We* numbers slightly decreased by 16.3 wt. % and 10.3 wt. %, while the *Oh* number increased by 13.1 wt. %, with the enhancement of H_2_O_2_ concentration from 8 wt. % to 35 wt. %, respectively. It resulted from the changes of comprehensive physical properties, including density, viscosity, and surface tension. Obviously, it was found that the increases in volume flow rate, nozzle size, and concentration resulted in the breakup becoming more difficult (Figure 6, Figure 9 and Figure 12) and producing larger droplet diameters (Figure 8, Figure 11, and Figure 13). Therefore, to improve the breakup performance, the effective surface tension needs to be minimized by applying a strong electric field.

When the H_2_O_2_ solutions suffer from an electric field, the electrification of H_2_O_2_ solutions is crucial to result in an effective reduction of the surface tension, contributing the fragmentation of the ligaments. The effective surface tension ($\gamma^{*}$) can be written as [30]:

(2) $$\gamma^{*}=\gamma -\frac{Q_{C}^{2}d_{i}^{3}}{48\varepsilon_{0}}$$

where *ε*_0_ is the vacuum permittivity (8.85 × 10^−12^ C^2^/(N·m^2^)), $Q_{c}=\sqrt{8\pi^{2}\varepsilon_{0}\gamma D^{3}}$ is the maximum volumetric charge density of the H_2_O_2_ solutions, in C/m^3^, and *D* is the droplet diameter.

Considering the effect of H_2_O_2_ concentration on the breakup behavior, the dimensionless parameters such as effective electrical Reynolds number (*Re_E_*(*φ*)), Weber number (*We_E_*(*φ*)), and Ohnesorge number (*Oh_E_*(*φ*)) can be expressed as [31]:

(3) $$Re_{E}(\varphi )=\frac{\rho (\varphi )b_{i}U}{\mu (\varphi )},We_{E}(\varphi )=\frac{\rho (\varphi )u^{2}d_{i}}{\gamma^{*}(\varphi )},Oh_{E}(\varphi )=\frac{\mu (\varphi )}{\sqrt{\rho (\varphi )\gamma^{*}(\varphi )d_{i}}}$$

where *b*_i_ is the ion mobility, *U* is the electric-field voltage. Owing to the Rayleigh limit, the H_2_O_2_ solution is partly electrified. It means that the charges of *q*(*φ*) are fractional to those of *Q_C_*(*φ*) [32]. Thus, the electrical dimensionless numbers can be calculated as:

(4) $$\left\{ \begin{array}{l} Re_{E}(\varphi )=\frac{\rho (\varphi )b_{i}U}{\mu (\varphi )}\\ We_{E}(\varphi )=\frac{\rho (\varphi )u^{2}d_{i}}{\gamma (\varphi )-q^{2}(\varphi )/\left(8\pi^{2}\varepsilon_{0}D^{3}\right)}\\ Oh_{E}(\varphi )=\frac{\mu (\varphi )}{\sqrt{\rho (\varphi )d_{i}(\gamma (\varphi )-q^{2}(\varphi )/(8\pi^{2}\varepsilon_{0}D^{3}))}} \end{array}\right.$$

Equation (4) suggests that the applied voltage and the electrification of the solution are crucial to influence the breakup performance of H_2_O_2_ ligaments. The nozzle size and the volume flow rate (related to the velocity) are another two operation conditions. Through applying a strong electric field, the effective surface tension obviously decreases since the term $‘Q_{C}^{2}(\varphi )d_{i}^{3}/48\varepsilon_{0}’$ in Equation (2) is much larger than zero, leading to an increase in *We_E_*(*φ*).

In the electric field, the ligament suffers from the gravity force, viscous force, electric-field force, atmospheric force, and surface tension. In the case of 28 kV, 21G, 500 mL/h, and 35 wt.%, the approximate calculations show that the order of the force magnitude (10^−5^~10^−4^ N) is the electric-field force, surface-tension force, gravity force, viscous force, and atmospheric force, respectively. It suggests that the electric-field force and surface-tension force play primary roles in influencing the breakup of the ligaments. The higher the applied electric-field voltage is, the more charges the ligaments obtain until saturation. If the electrostatic force is larger than the surface-tension force, the ligaments become unstable and undergo catastrophic fragmentation.

The H_2_O_2_ concentration has a significant effect on the physical properties that comprehensively influence the breakup performance of H_2_O_2_ ligaments. For the case of 8~35 wt. % concentration, although the difference of viscosity of 10% is greater than the difference of surface tension of 1.4%, the magnitude of the electric-field force and surface tension force is larger than the viscous force. It indicates that the electric field and the surface tension forces mainly contribute to the breakup of the ligaments.

With increasing H_2_O_2_ concentration, the volumetric charge density *Q_C_*(*φ*) and the surface-tension coefficient γ(φ) simultaneously change. In our previous work [27], the critical concentration and minimum effective surface tension can be determined using the following equation:

(5) $$\gamma^{*}(\varphi_{c})=Min\left[\gamma (\varphi )-\frac{Q_{C}^{2}(\varphi )d_{i}^{3}}{48\varepsilon_{0}}\right],if\frac{d\gamma^{*}(\varphi )}{d\varphi }=0\;and\frac{d^{2}\gamma^{*}(\varphi )}{d\varphi^{2}}>0.$$

According to Equation (5), for this case of 8~35 wt. % concentration, the effective surface tension gradually increases with the increase in the concentration. Thus, as the H_2_O_2_ concentration is 8 wt. %, the effective surface tension is minimum, resulting in the smallest breakup droplet diameter. The calculation is consistent with the experimental one shown in Figure 13. For the H_2_O_2_ solution with the concentration of 8 wt. %, if the nozzle size of 0.5 mm is installed, the optimum operation conditions keep the supply of the volume flow rate of 500 mL/h at the voltage of 28 kV.

## 4. Conclusions

Experiments on the atomization characteristics of H_2_O_2_ solutions in an electrostatic field were performed. The effects of applied electric-field voltage, nozzle size, volume flow rate, and H_2_O_2_ concentration on electrostatic breakup performance were evaluated in detail. The breakup mechanism was deeply discussed by the analysis of dimensionless parameters.

The increase in applied electric-field voltage, reduction in the nozzle size, and modulation of the volume flow rate significantly improve the breakup performance, producing finer and more uniformly distributed droplets. For H_2_O_2_ solution of 35 wt. % concentration, the smallest average diameter of breakup droplets reached 92.8 μm at the operation conditions of 28 kV, 0.5 mm, and 500 mL/h, respectively. With decreasing H_2_O_2_ concentration (8~35 wt. %), at optimum operation conditions, the average diameters of breakup droplets decreased. For H_2_O_2_ solution of 8 wt. % concentration, the average diameter was 67.4 μm. The calculation on the concentration effect on breakup performance shows that the average diameter of breakup droplets reaches its minimum at 8 wt. % concentration, which is consistent with the experimental results. To obtain the best atomization performance, the operation conditions and physical properties need to be modulated for practical industrial application.

The electrostatic atomization of H_2_O_2_ was carried out at room temperature and atmospheric pressure, no water vapor was produced, and then the water-condensation disadvantage could be eliminated during the sterilization process as well as in the propulsion system. Moreover, the electrostatic atomization of H_2_O_2_ makes it easy to be ready to use, which fits for the miniaturization of the propulsion system to meet with the continuing demand to develop the capabilities of micro-/nanosatellites.

## Figures and Tables

**Table 1:** The correlations of density, surface tension, viscosity, and permittivity of H_2_O_2_ solutions with the concentration.

Physical Properties	Model Formula
Density (*ρ*)	ρ=0.0044φ+0.9846; R^2^ = 0.994
Viscosity (*μ*)	$\mu =-6.64891\times 10^{-7}\varphi^{3}+8.13872\times 10^{-5}\varphi^{2}+9.81670\times 10^{-4}\varphi +1.00178$; R^2^ = 0.998
Surface tension (*γ*)	$\gamma =4.55974\times 10^{-4}\varphi^{2}+0.03170\varphi +72.69847$; R^2^ = 0.994
Permittivity (*ε*)	$\varepsilon =-3.17113\times 10^{-5}\varphi^{3}+0.0021\varphi^{2}+0.03261\varphi +80.45818$; R^2^ = 0.990

**Table 2:** Dimensions of atomization nozzles.

Nozzle Types	Inner Diameter (*d*_i_, mm)	Outer Diameter (*d*_o_, mm)
10G	3.00	3.50
11G	2.50	3.00
12G	2.20	2.80
13G	1.90	2.40
14G	1.55	2.10
15G	1.35	1.80
16G	1.16	1.60
17G	1.07	1.47
18G	0.86	1.26
19G	0.67	1.07
20G	0.60	0.90
21G	0.50	0.80
22G	0.40	0.70
23G	0.33	0.63
24G	0.30	0.55
25G	0.26	0.50
26G	0.24	0.45
27G	0.21	0.40
28G	0.19	0.35
30G	0.16	0.31
